# Beyond NIMBYs and NOOMBYs: what can wind farm controversies teach us about public involvement in hospital closures?

**DOI:** 10.1186/s12913-015-1172-x

**Published:** 2015-12-01

**Authors:** Ellen Stewart, Mhairi Aitken

**Affiliations:** Centre for Population Health Sciences, University of Edinburgh, Edinburgh, EH8 9AG UK

**Keywords:** Service change, Public participation, Public protest, Wind energy, Public engagement

## Abstract

**Background:**

Many policymakers, researchers and commentators argue that hospital closures are necessary as health systems adapt to new technological and financial contexts, and as population health needs in developed countries shift. However closures are often unpopular with local communities. Previous research has characterised public opposition as an obstacle to change. Public opposition to the siting of wind farms, often described as NIMBYism (Not In My Back Yard), is a useful comparator issue to the perceived NOOMBYism (Not Out Of My Back Yard) of hospital closure protestors.

**Discussion:**

The analysis of public attitudes to wind farms has moved from a fairly crude characterisation of the ‘attitude-behaviour gap’ between publics who support the idea of wind energy, but oppose local wind farms, to empirical, often qualitative, studies of public perspectives. These have emphasised the complexity of public attitudes, and revealed some of the ‘rational’ concerns which lie beneath protests. Research has also explored processes of community engagement within the wind farm decision-making process, and the crucial role of trust between communities, authorities, and developers.

**Summary:**

Drawing on what has been learnt from studies of opposition to wind farms, we suggest a range of questions and approaches to explore public perspectives on hospital closure more thoroughly. Understanding the range of public responses to service change is an important first step in resolving the practical dilemma of effecting health system transformation in a democratic fashion.

## Background

### Public responses to hospital closures

Proposals to close hospitals are among the most politicised decisions in health-care, sitting at the intersection of three major contemporary trends; disinvestment, evidence-based policy, and public involvement. The drivers for closures vary, but include major international trends such as the move towards large, centralised hospitals providing full ranges of services, concentrating clinical expertise and entailing the closure of numbers of smaller, local hospitals [1], and the aftermath of the financial crisis [2, 3]. Historically, changing patterns of health care, such as deinstitutionalisation within psychiatric services, have prompted closures [4, 5]. Within tax-funded, state-managed health systems decisions about closure rely on a fragile constellation of actors including democratically-elected politicians, health service managers, staff, unions and professional bodies, and, of course, the public. This article focuses on public responses to hospital closures, proposing that both policy debate and much health services research in this area could learn valuable lessons from literature on public opposition to the siting of wind farms. We begin by arguing that the analysis of public opposition to closure lacks an empirical base, before outlining a rationale for the comparison with opposition to wind farms. The discussion section briefly summarises the development of the literature on wind farms, before distilling two key messages for the analysis of hospital closure controversies. Drawing on these, our concluding section suggests a range of questions and approaches which might prompt a more fruitful analysis of public opposition to closures in the future.

Researchers often cite public opposition as a critical factor in the success or failure of disinvestment processes [6, 7], and yet until very recently [8] there has been little sustained academic engagement with the nature and dynamics of this opposition. This has not prevented authors from making bold assertions about its extent and characteristics within studies which report no data collection with members of the public (see for example [9]). While research often mentions public opposition, few studies involve empirical data collection with anti-closure campaigners or other members of the public. Even where relatively nuanced accounts of conflict have been produced (often by geographers), media coverage, official documents, or interviews with health service managers or elected politicians are used as a proxy for public perspectives [6, 10–13].

Policymakers and commentators have been similarly reluctant to engage with closure opposition in a meaningful way: in a recent project on service reconfiguration, Fulop, Walters et al. [6] argue that national policy in England assumes that sufficiently communicating the ‘right’ evidence will change public opinion. The English Department of Health has been urged to ‘grasp the nettle’ of unpopular closures ([14], see also [15]). In Canada, where several provinces closed substantial numbers of hospitals in the 1990s, key actors are reported reflecting that “drastic change is only achieved by strong external forces” [16]; another report lists “overcoming institutional and local loyalties” as a vital route to change [17]. While the difficult and slow process of closure is undoubtedly frustrating to those convinced of the clinical and financial evidence base for change, characterising public opposition as a ‘nettle to be grasped’ and (democratic) political oversight as a wrinkle to be ironed out of an otherwise smooth process locates actors within a highly technocratic perspective, and risks making unfounded assumptions about public perspectives on hospital closures.

In a recent invited commentary for Social Science & Medicine, Cohen and Ahern [18] drew on published evidence on school closures to encourage researchers examining hospital closure to move beyond quantitative studies and “elucidate some of the nuances and unanswered questions, including how public hospital closure decisions are made and the positions of the different stakeholders involved”. Other authors have explored parallels between rural maternity reconfigurations and community responses to large-scale supermarket developments, with the intention of better understanding the wider context of health services, and public expectations of them [19]. Here, we propose another instructive avenue of comparison; the evolution of research on opposition to wind energy developments. This article draws on this substantial literature to distil several key messages for a more thoroughgoing, and democratically-minded, engagement with public perspectives on the vexed issue of hospital closures within tax-funded, state-managed health systems. This debate article reviews existing published research and did not involve any primary data collection with human subjects: accordingly no ethical approval was required and there is no supporting data to make available.

## Why compare hospital closure and windfarm controversies?

Specific lessons from this literature are described in the next section, but the rationale for the comparison between wind farm siting and hospital closure is worth elucidating. Health researchers often emphasise the exceptionalism of their subject area, which is intimately concerned with matters of life and death, and where information asymmetries between providers and publics are substantial [20]. However our understanding of health services in their social context can be strengthened by looking at the other services and facilities located in communities. Rather than understanding conflict over decisions about wind farms and hospital closures as the implementation of rational policy goals gone awry, models of policy analysis such as that offered by Stone understand conflict and negotiation as an intrinsic part all stages of the policy process [21]. Both wind farm development and hospital closures are issues of public concern where policymakers describe a perceived mis-match between what is politically desirable and politically feasible. A significant evidence base suggests that both centralised hospital services and wind energy respectively will bring societal benefits, but these are based on technical, expert knowledge which is difficult to communicate, and, crucially, difficult for local populations to engage with in order to understand or contest it. At the level of implementation, opposition has centred on the perceived local costs to be borne. In both cases, national (even international) agendas drive visible changes to the local environment, whether adding wind turbines or demolishing or changing the use of prominent buildings. These changes impact on daily life in these communities - including the employment of local people and the social role of public spaces – beyond the specific functions of the facility.

Both advocates of wind energy and advocates of healthcare modernisation express frustration at populations who resist local changes. This often takes the form of accusations of rationally indefensible ‘NIMBYism’ (Not In My Back Yard-ism), or, in the case of opposition to hospital closures, what has been termed ‘NOOMBYism’ (Not Out Of My Back Yard-ism), on the part of publics. The ‘NIMBY syndrome’ was a term coined in 1970s USA, in the particular context of conflict over the siting of hazardous waste facilities, but it has become a familiar accusation in both academic and popular debates about decisions on a much wider range of locational dilemmas [22]. The counterpart NOOMBYism (sometimes described as IMBYism: In My Back Yard-ism) has more occasionally cropped up in popular debates on planning issues which involve the removal of services or facilities, and in academic papers [23, 24]. In both cases, these terms are pejorative descriptions which suggest frustration at, rather than engagement with, the complexity of public perspectives on the locally-experienced impact of agendas for change which are formulated and justified elsewhere. However while health policy debates seem entrenched in this position, we argue that the increasingly interdisciplinary literature around public responses to wind power has managed to progress beyond this point, via a thoroughgoing empirical engagement with public experiences, as opposed to assumed or imagined public perspectives.

## Discussion

### Lessons from windfarm controversies

Localised public opposition to onshore wind farms has frequently been pointed to as an obstacle – or at least a challenge – for the realisation of renewable energy deployment targets [25–30]. Whilst onshore wind farms are the most mature renewable energy technology currently available and largely viewed as market ready [31] their deployment has stalled. There are a number of factors contributing to this [32]; however researchers have paid significant attention to local community opposition to proposed developments which they describe as causing a “bottleneck” in the planning system [30]. In this section, we review the literature on public opposition to wind farms as it has evolved over recent years, and distill two key ‘lessons’ with relevance for policy and research in the area of hospital closures.

The starting point for much literature on public opposition to wind farms has been a perceived dissonance between high levels of public support for renewable energy (and wind power in particular) but simultaneously frequent localised opposition to particular proposed wind power developments [28, 33]. Numerous studies have set out to explain this ‘gap’ between high general levels of support for wind power (reported in opinion polls and surveys) and localised opposition to particular developments.

As a consequence, until recently much of the literature has been focussed at understanding opposition (rather than public experiences or responses more broadly) [30] as ‘deviant’ from normal public opinion [25], and explaining what is described as an ‘attitude–behaviour gap’ amongst the public [30]. The vision of the public as contradictory and irrational has informed managerial approaches to the study of public responses to wind power, which have sought to understand public opposition in order to overcome or avoid it in the future (rather than meaningfully engage with objectors and/or address the basis of their concern).

NIMBY explanations of public opposition to wind farms represent a prominent example of such approaches. According to NIMBY explanations individuals may support wind power in the abstract (and at distant locations [33]), but oppose wind power projects in their locale [34]. NIMBY explanations have been a pervasive and persuasive force within academic and policy literatures relating to wind power [30] and the term has entered the vocabulary of actors involved in wind power controversies. Burningham [35] notes that the language of NIMBYism is widely used by parties involved in planning conflicts. Both Barry et al. [26] and van der Horst [36] note that opponents to wind power developments are often aware of the potential to be branded a “NIMBY” and seek to avoid being portrayed as such. The evidence base on public opposition to wind farms has grown rapidly in the last 15 years, and its development offers two key messages for the analysis of controversial hospital closures.

### Message 1: empirical research on the nuances of support and opposition is essential

Despite the prevalence of NIMBY explanations in policy and ‘real world’ debates, these have come to be widely discredited in the academic literature. A series of studies has highlighted complexity and nuance within the perceived monoliths of both general public opinion on wind power and on specific instances of opposition to wind farm siting. The underlying assumption that the majority of the public support wind power has been questioned. The literature has typically cited opinion polls and surveys to demonstrate this high support but takes an uncritical and positivist approach to this data [37]. This means that important considerations such as who commissioned polls and for what purpose; how questions were framed; or how the sample was selected are overlooked. It has also at times taken a selective approach to the reporting of opinion poll data to strengthen the conviction that the majority of the public support wind power [25]. Ellis et al. [30] contend that ‘the most popularly deployed methodology, the opinion poll, has contributed to the impasse in understanding public perception of wind farms’.

Following critical reviews of the literature, recent years have witnessed a shift in approach and a rise in qualitative studies exploring the nuances and realities of public opposition and support in much more depth. For example, Wolsink has argued that NIMBY explanations neglect the true diversity of motivations for public opposition to wind power developments [33]. Similarly, Devine-Wright [38] points to various ‘independent variables’ which influence how perceptions of wind power developments are perceived. The existence of such complex variables – which ‘include physical, contextual, political, socio-economic, social, local and personal aspects and reflect the complex, multidimensional nature of forces shaping public perception’ [38] - highlights the inadequacy of NIMBY explanations.

This growing body of literature points to the complexities of public opinions [27]; the importance of considering local values and contextual factors [38]; the considerable value of local knowledge and experience [37]; and the multiple forms that opposition to wind farms can take [39]. In doing so it has highlighted the limited utility of managerial approaches to addressing public opposition.

Devine-Wright [38] argues that qualitative methods are best suited to investigating representations of wind turbines in “different social groups, within and across communities’. The literature now emphasises the importance of qualitative methods for understanding how opinions change over time and how geographical, temporal, socio-political or cultural contexts influence and alter public responses (as has been demonstrated by [40, 41]). Public opinion is recognised as being ‘highly flexible, transitory and adaptable’ [25].

### Message 2: pay attention to processes of engagement, not just outcomes

Academic and policy literatures relating to planning of renewable energy projects place increasing emphasis on the importance and value of community engagement. A central theme to emerge through this literature is the importance of trust: trust built up in planning and pre-planning processes can lead to increased levels of support for proposed developments and developers [42]. As Gross [43] has observed, perceived fairness of outcomes appears to be inextricably linked to perceived fairness of processes. Seeking to understand opposition simply to overcome or avoid it does little to engender trust. Such instrumental approaches to community engagement can cause considerable harm to developer/planner-community relationships and in many cases are a contributing factor to the emergence or crystallisation of local opposition [42]. Wynne [44] cautions that those conducting community engagement should not expect participants ‘to trust oneself, if one’s assumed objective is to manage and control [their] response’.

Therefore, the literature relating to public responses to wind farms now demonstrates a consensus around the importance of effective community engagement to understand and address local concerns, values and/or priorities.

## Implications for analysis of hospital closures

The example of public opposition to wind farms demonstrates some of the challenges in researching issues which are both topical and appear to present intractable dilemmas for policymakers. External pressure to solve a problem might not always be conducive to opening up an issue. However in a relatively short number of years, the initial problem-solving, managerial approach outlined above has largely given way to an interdisciplinary critique which seeks to problematize, engage with and unpack the apparently ‘NIMBY’ attitudes of public protestors. The issue of public opposition to hospital closures similarly has the potential to generate ‘heat’, and yet in decades of study there has been far less progression beyond querying how we can overcome ‘irrational’ public opposition to hospital closures.

This is likely partly attributable to the respective research contexts. Health services research, although a diverse and multidisciplinary field remains heavily influenced by the positivistic epistemological traditions which ground the biomedical sciences. Furthermore the prevalence of heavily applied externally-funded research (often commissioned to assess proposed or actual changes in policy [45]) and vocal thinktanks who conduct research in order to influence policy and practice, may limit the scope for more considered theoretical reflection. By contrast the highly multi- and interdisciplinary literature relating to public responses to wind power development has generated significant debate and exchange of ideas, enabling it to evolve relatively quickly.

Learning from related issues where ‘experts’ find themselves confronting critical publics, what might a renewed analysis of public responses to hospital closures entail? For researchers, the imperative to engage empirically with public perspectives points us not just to different methods, but also to different questions. In contrast to the wealth of public opinion data on wind energy in general, little is known about how the general public feels about new, centralised models of healthcare. Beyond asking what ‘the’ (monolithic) public or community thinks on an issue we might look for clusters and patterns of perspective at the local level. How widespread is opposition to closures? It might be that campaigns are restricted to small vocal pockets who have particularly strong stakes in a particular element of a service reconfiguration. Goyder [46] sought to establish whether protestors who signed an anti-closure petition really did have stronger views on the issue than the general population, or whether they were “merely those shanghaied into adding their name to a list”. Who are the key actors, and how do they self-identify? Much of the existing research which describes campaigns struggles to distinguish staff at threatened facilities from members of the public [47, 48], particularly in the literature on closures in rural areas [49]. The extent to which campaigns are initiated by [50] or understand themselves as representing particular groups is salient to understanding their perspectives. Relatedly, who isn’t engaged? Authors often attempt to convey strength of opposition via numerical accounts of petition signers or march attendees, often as a proportion of the local population [13, 51, 52]. As well as the difficulty of verifying such figures, it is difficult to make sense of the absences from such collective upsurges.

The suggestion that we research processes, and not merely outcomes, of engagement, implies that both researchers and policy commentators engage more thoroughly, and less disparagingly, with the political nature of health service decision-making. Quantitative studies of how rational closures are [9] or indeed of how many lives they save [53] have their place, but they do not negate the presence of opposition, nor render it intrinsically illegitimate. Moving away from a ‘deficit’ model of public understandings of science, towards one which acknowledges the possibility of different knowledge bases, entails attending to how authorities listen to and negotiate with opposed publics, beyond the “decide-announce-defend” model critiqued in studies of energy politics [29]. Social scientific research can explore the role of generalised relationships of (mis)trust between publics and their health systems, as the context in which particular decisions take on specific meanings for local populations. Multiple studies note the repeated introduction of proposals to close a facility over a number of years [13, 51, 54, 55], and yet there is little attempt to explore the impact of this longitudinally within a local population. Attending to process, rather than outcome, might also yield more optimistic findings. Current studies tend to start from the point of mobilisation against a proposal, but it remains unclear whether cases of negligible protest [6, 56] are simply intrinsically less contentious, or whether a well-conducted process of public engagement can square the circle of NOOMBY opposition to apparently essential reconfiguration.

## Conclusions

This article argues that current research and policy debates on public responses to hospital closures rely too heavily on assumptions about the irrationality of a perceived singular public view. While highly visible rallies and campaigns (purposely) present a simple message of opposition, there is scope for a much more thoroughgoing analysis of public perspectives on, and opposition to, ‘evidence-based’ policy goals. Engaging with disagreement as an intrinsic part of the policy process, rather than ignoring or dismissing it, can acknowledge its creative role in moving towards collectively acceptable public outcomes [21, 57]. The literature relating to public responses to wind power points to the benefits of engaging with diverse research and methodological approaches. The evidence base has evolved considerably over the past ten to 15 years through a reflexive process of critique, which has drawn attention to previous shortcomings and limitations. Notably, the literature in this area has moved beyond simplistic characterisations of public opposition and towards nuanced, qualitative studies which grapple with the complexities of public opinions and responses. Such studies have acknowledged the importance of aiming to understand, rather than simply manage or overcome public responses. This enthusiasm for, and professed commitment to community engagement does not easily or predictably translate into meaningful engagement in practice. The language of NIMBYism is alive and well in everyday conflicts over wind farm developments [26, 36] and objectors to proposed developments continue to be routinely discredited as NIMBYs in media coverage and popular discourse. Whilst examples of good practice do exist, community engagement conducted by wind farm developers often falls short of the considered approaches advocated in the academic literature [42]. Nonetheless there are valuable lessons within the energy politics literature for the analysis of public responses to hospital closures. Improving our understanding of these responses is a crucial step in moving beyond the current impasse.

## Abbreviations

NIMBY: ‘Not in my back yard’
NOOMBY: ‘Not out of my back yard’
